# Ageing and dementia in low and middle income countries - Using research to engage with public and policy makers

**DOI:** 10.1080/09540260802094712

**Published:** 2008-10-16

**Authors:** Martin Prince, Daisy Acosta, Emiliano Albanese, Raul Arizaga, Cleusa P. Ferri, Mariella Guerra, Yueqin Huang, Ks Jacob, Ivonne Z. Jimenez-Velazquez, Juan Llibre Rodriguez, Aquiles Salas, Ana Luisa Sosa, Renata Sousa, Richard Uwakwe, Rikus Van Der Poel, Joseph Williams, Marc Wortmann

**Affiliations:** ^1^Section of Epidemiology, Health Services Research, King’s College London, London, UK; ^2^Internal Medicine Department, Geriatric Section Universidad Nacional Pedro Henriquez Ureña (UNPHU), Santo DomingoDominican Republic; ^3^Behavioral and Cognitive Neurology Unit, Neuraxis Institute - Neurological Foundation, Buenos Aires – Argentina; ^4^Psychogeriatric Unit, National Institute of Mental Health “Honorio Delgado Hideyo Noguchi”, Lima – Perú; ^5^Institute of Mental Health, Peking University, Haidian District Beijing, China; ^6^Christian Medical College, Vellore, India; ^7^University of Puerto Rico, School of Medicine, San Juan, Puerto Rico; ^8^Facultad de Medicina Finley-Albarran, Medical University of Havana, Cuba; ^9^Medicine Department, Caracas University Hospital, Faculty of Medicine, Universidad Central de Venezuela, Caracas; ^10^The Cognition and Behavior Unit, National Institute of Neurology and Neurosurgery of Mexico, Mexico City, Mexico; ^11^Dept. of Mental Health, Nnamdi Azikiwe University Teaching Hospital, Nnewi, Anambra State, Nigeria; ^12^University of the Free State Bloemfontein, South Africa; ^13^Department of Community Health, Voluntary Health Services, Chennai, India; ^14^Executive Director, Alzheimer’s Disease International, London, UK

## Abstract

Abstract While two thirds of the 24 million people with dementia worldwide live in low and middle income countries, very little research has been conducted to support policy making in these regions. Among the non-communicable diseases, dementia (in common with other chronic NCDs linked more to long-term disability than to mortality) has been relatively under-prioritized. International agreements, plans and policy guidelines have called for an end to ageist discrimination and a focus upon reducing disadvantage arising from poverty and the consequences of ill health. Social protection, access to good quality age-appropriate healthcare and addressing the problem of disability are all key issues. However, as yet, little progress has been made in addressing these concerns. In this review we outline the current international policy agenda for older individuals, and its specific relevance to those with dementia and other disabling non-communicable diseases. We consider the potential for epidemiological research to raise awareness, refine the policy agenda, and promote action, using the example of the dissemination strategy developed by the 10/66 Dementia Research Group.

## Introduction

The 10/66 Dementia Research Group is so named to draw attention to the relative neglect of people with dementia living in developing countries by the global research community – just 10% of the research focused upon the two thirds of those with dementia living in low and middle income countries (LAMIC). In the 10 years since its foundation in 1998, the group has:

1. Developed an innovative approach to culture and education-fair diagnosis (Prince, Acosta, Chiu, Scazufca, & Varghese, 2003).
2. Published some of the first accounts of care arrangements and caregiver strain, from pilot data in 26 LAMIC centres (Choo, et al., 2003; Dias et al., 2004; Ferri, Ames, & Prince, 2004; Patel & Prince, 2001; Prince, 2004; Shaji, Smitha, Praveen Lal & Prince, 2002b).
3. Worked with Alzheimer’s Disease International (ADI) and other international experts to estimate the numbers of people with dementia in all world regions and trends to 2040 (Ferri et al., 2005).

In this paper, we review the need for more research, particularly descriptive research to raise awareness and inform health and social policy. We review existing international agreements and policy guidelines, and the rights and policy priorities that they have established for older people living in LAMIC. Finally, we consider the relevance of the new 10/66 population-based studies (Prince et al., 2007a) to this policy agenda, and the ways in which this, and other new evidence can be used both to refine the agenda and promote action.

## The need for more research

In poor but rapidly developing regions of the world people are living longer, and having fewer children. High fat diets, smoking and sedentary lifestyles are becoming more common. Chronic non-communicable diseases (NCDs) linked to ageing – heart disease, stroke, cancer and dementia – are much more in evidence, and beginning to be recognized as a public health priority (Fuster & Voute, 2005). While cancer and heart disease contribute mainly to mortality, much of the burden of other NCDs (dementia and mental disorders diabetes and stroke) arises from years lived with disability (WHO, 2006). These are under-prioritized conditions with respect to research, policy and practice. Dementia, which has a uniquely devastating impact on capacity for independent living is often forgotten when policies for prevention and treatment of NCDs are proposed, as for example with the recent Lancet series on non-communicable diseases (Strong, Mathers, Leeder, & Beaglehole, 2005) and the WHO’s Global Report on Innovative Care for Chronic Conditions (Epping-Jordan, Pruitt, Bengoa, & Wagner, 2004).

24.2 million people live with dementia worldwide, with 4.6 million new cases annually (Ferri et al., 2005) (similar to the global incidence of non-fatal stroke (Mackay & Mensah, 2004)). Two thirds live in LAMIC. Numbers will double every twenty years to over 80 million by 2040, with much sharper increases in developing than developed regions. These are provisional estimates, given that prevalence data are lacking in many world regions, and patchy in others, with few studies and widely varying estimates (Ferri et al., 2005). Coverage is good in Europe, North America, and in developed Asia-Pacific countries; South Korea, Japan, Taiwan and Australia. Several studies have been published from India and China, but estimates are too few and/or too variable to provide a consistent overview for these huge countries. There is a particular dearth of published epidemiological studies in Latin America (with just two studies from Brazil (Herrera, Caramelli, Silveira, & Nitrini, 2002; Nitrini et al., 2004) and one from Colombia (Rosselli et al., 2000)), Africa (with just one study, from Nigeria (Hendrie et al., 1995)), Russia, the Middle East and Indonesia.

Worldwide, surprisingly few epidemiological studies of dementia have gone beyond reporting on prevalence, incidence and aetiology. The impact of dementia upon the individual, the family and society has been little studied, particularly its contribution to disability, dependency, caregiver strain and costs. The response of health services and systems has also been relatively neglected.

The research community has an important role to play in engaging the attention of public and policy makers. Evidence from population-based research on ageing and dementia from low and middle income countries (LAMIC) should help to stimulate a wider debate about older people’s health and social care needs, and how they should be met. Good quality research, effectively disseminated, can raise awareness, inform evidence-based policy making, and service development.

## International treaties, policies and plans

International treaties and policy documents have set out an agenda comprising on the one hand universal rights, and on the other objectives and actions of specific relevance to the needs of older people. Much of the focus is upon vulnerability, particularly that arising from disabling health conditions, including dementia. There seems little doubt that their full implementation would have led to improvements in health, well-being and quality of life. Resource limitations are evidently a major obstacle, particularly for poor countries. Inaction may also be explained by lack of awareness of recommendations, and lack of appropriate prioritization of the needs of these marginalized groups. Data from epidemio-logical research can remind governments of their obligations, provide evidence of the extent to which these have, or have not been met, and hence assist stakeholders in holding governments to account.

### UN charters, conventions and plans

These provide a framework of rights and responsibilities which, in the case of charters and conventions, once ratified, are binding in international law. For older people the most relevant instruments are:

#### The Universal Declaration of Human Rights (1948)

Article 25(1) states ‘Everyone has the right to a standard of living adequate for the health and well-being of himself and of his family, including food, clothing, housing and medical care and necessary social services, and the right to security in the event of unemployment, sickness, disability, widowhood, old age or other lack of livelihood in circumstances beyond his control’.

#### The UN Convention on the Rights of Persons with Disabilities (2006)

Article 1 requires governments ‘to promote, protect and ensure the full and equal enjoyment of all human rights and fundamental freedoms by all persons with disabilities, and to promote respect for their inherent dignity’. Of particular relevance to those with dementia are the right to live in the community, the right to an adequate income, and the right to health (including a specific requirement that the Government must increase the availability of quality and affordable healthcare for individuals with disabilities).

#### The Madrid International Plan of Action on Ageing (Madrid Plan) (United Nations, 2002)

The Madrid Plan adopted by 159 nations at the United Nations Second World Assembly on Ageing, Madrid 2002, does not carry the same force in international law as a UN treaty, but does set out detailed objectives with accompanying actions to respond to the challenges of population ageing. Ten objectives are particularly relevant to the needs of people with dementia. These relate to the needs

- for intergenerational solidarity
- to address poverty, providing income security and social protection for older people
- for affordable, accessible age-appropriate health care
- to address the problem of disability

To implement the plan the Assembly called for

- ‘harnessing of research ... to focus on the individual, social and health implications of ageing, in particular in developing countries’;
- ‘facilitating partnership between all levels of government, civil society, the private sector and older persons’;
- ‘recognition of the situation of ageing persons, their unique circumstances and the need to give them an effective voice in decisions affecting them’.

### World Health Organization policy recommendations

The World Health Organization often works with international technical experts to develop policy and practice guidelines for governments. While in no way binding, these detailed documents can be influential in steering national, regional and global health policy.

1. The WHO policy document *Towards an international consensus on policy for long-term care of the ageing* (WHO, 2000) describes principles to inform policies for sustainable programmes in long-term care that are consistent with the priorities of countries at different levels of development, as a first step towards devising an international consensus.
2. The WHO Global Report on *Innovative care for chronic conditions* (WHO, 2002) alerts policy makers, particularly those in LAMIC to the implications of the decreases in communicable diseases and the rapid ageing of populations – healthcare is currently organized around an acute, episodic model of care that no longer meets the needs of many patients, especially those with chronic conditions. The WHO Innovative Care for Chronic Conditions framework (Epping-Jordan et al., 2004), provides a flexible and comprehensive basis on which to build or redesign health systems that are fit for purpose at the micro (patient and family), meso (healthcare organization and community), and macro (policy) levels.

### Alzheimer’s Disease International’s Kyoto Declaration

At their 20th annual conference held in Kyoto, Japan, ADI released a Kyoto Declaration, comprising minimum recommendations for dementia care, based upon overall recommendations from the 2001 WHO Health Report. The ADI Kyoto Declaration benchmarks progress in ten key areas (see Table I). The framework addresses health services and system structures, treatment gaps, policies, research and training and identifies target levels of attainment, for countries with low, medium and high levels of resources. Hence, it proposes a feasible, pragmatic series of objectives and actions for health systems at all levels of development.

**Table I:** Minimum actions required for dementia care (based on overall recommendations from the World Health Report, 2001).

Ten overall recommendations	Scenario A: Countries with a low level of resources	Scenario B: Countries with a medium level of resources	Scenario C: Countries with a high level of resources
1. Provide treatment in primary care	Recognize dementia care as a component of primary healthcare	Develop locally relevant training materials	Improve effectiveness of management of dementia in primary health care
	Include the recognition and treatment of dementia in training curricula of all health personnel	Provide refresher training to primary care physicians (100% coverage in 5 years)	Improve referral patterns
	Provide refresher training to primary care physicians (at least 50% coverage in 5 years).		
2. Make appropriate treatments available	Increase availability of essential drugs for the treatment of dementia and associated psychological and behavioural symptoms.	Ensure availability of essential drugs in all healthcare settings.	Provide easier access to newer drugs (eg. anticholinesterase agents) under public or private treatment plans
	Develop and evaluate basic educational and training interventions for caregivers	Make effective caregiver interventions generally available	
3. Give care in the community	Establish the principle that people with dementia are best assessed and treated in their own homes	Initiate pilot projects on integration of dementia care with general healthcare	Develop alternative residential facilities
	Develop and promote standard needs assessments for use in primary and secondary care	Provide community care facilities (at least 50% coverage with multidisciplinary community teams, day care, respite and inpatient units for acute assessment and treatment)	Provide community care facilities (100% coverage)
	Initiate pilot projects on development of multidisciplinary community care teams, day care and short-term respite	According to need, encourage the development of residential and nursing home facilities, including regulatory framework and system for staff training and accreditation	Give individualized care in the community to people with dementia
	Move people with dementia out of inappropriate institutional settings		
4. Educate the public	Promote public campaigns against stigma and discrimination	Use the mass media to promote awareness of dementia, foster positive attitudes, and help prevent cognitive impairment and dementia	Launch public campaigns for early help-seeking, recognition and appropriate management of dementia
	Support non-governmental organizations in public education		
5. Involve communities, families and consumers	Support the formation of self-help groups	Ensure representation of communities, families, and consumers in policy-making, service development and implementation	Foster advocacy initiatives
	Fund schemes for non-governmental organizations		
6. Establish national policies, programmes and legislation	Revise legislation based on current knowledge and human rights considerations	Implement dementia care policies at national and subnational levels	Ensure fairness in access to primary and secondary health care services, and to social welfare programmes and benefits
	Formulate dementia care programmes and poicies – Legal framework to support and protect those with impaired mental capacity – Inclusion of people with dementia in disability benefit schemes – Inclusion of caregivers in compensatory benefit schemes	Establish health and social care budgets for dementia care	
	Establish health and social care budgets for older persons	Increase the budget for mental health care	
7. Develop human resources	Train primary healthcare workers.	Create a network of national training centres for physicians, psychiatrists, nurses, psychologists and social workers	Train specialists in advanced treatment skills
	Initiate higher professional training programmes for doctors and nurses in old age psychiatry and medicine.		
	Develop training and resource centres		
8. Link with other sectors	Initiate community, school and workplace dementia awareness programmes	Strengthen community programmes	Occupational health services for people with early dementia
	Encourage the activities of non-governmental organizations		Provide special facilities in the workplace for caregivers of people with dementia.
			Initiate evidence-based mental health promotion programmes in collaboration with other sectors
9. Monitor community health	Include dementia in basic health information systems	Institute surveillance for early dementia in the community	Develop advanced monitoring systems
	Survey high-risk population groups		Monitor effectiveness of preventive programmes
10. Support more research	Conduct studies in primary healthcare settings on the prevalence, course, outcome and impact of dementia in the community	Institute effectiveness and cost-effectiveness studies for community management of dementia	Extend research on the causes of dementia
			Carry out research on service delivery
			Investigate evidence on the prevention of dementia

## Research into policy

### The 10/66 evidence base

The 10/66 Dementia Research Group has conducted population-based surveys (2003–2007) of dementia prevalence and impact in 14 catchment areas in 10 low and middle income countries (India, China, Nigeria, Cuba, Dominican Republic, Brazil, Venezuela, Mexico, Peru and Argentina) (Prince et al., 2007a). New studies are also underway in Puerto Rico and South Africa. China, India, Peru, Mexico and Argentina recruited from separate urban and rural catchment areas; the other centres included urban catchment areas only. Cross-sectional comprehensive one phase surveys have been conducted of all residents aged 65 and over of geographically defined catchment areas in each centre with a sample size of between 1,000 and 3,000 (generally 2,000) in each of the ten countries. Each of the studies uses the same core minimum data set with cross-culturally validated assessments (dementia diagnosis and subtypes, mental disorders, physical health, anthropometry, demographics, extensive non-communicable disease risk factor questionnaires, disability/ functioning, health service utilization, care arrangements and caregiver strain). The net result will be a unique resource of directly comparable data, comprising 19,000 older adults from three continents. A publicly accessible data archive will be established as a resource for the academic and policy community. Nested within the population-based studies is a randomized controlled trial of a caregiver intervention for people with dementia and their families. We will shortly embark on an incidence phase with a 2.5 to three year follow-up of baseline participants in seven of the 10 countries (2007–2010).

## Priority areas

The 10/66 Dementia Research Group developed its dissemination strategy at a Bellagio workshop in 2006, funded by the Rockefeller Foundation. We have subsequently identified four key areas for evidence-based policy development, consistent with the internationally agreed priorities outlined above and supported by the initial evidence from our pilot and population-based research programmes.

### Increasing awareness

Alzheimer’s Disease International has identified raising awareness of dementia among the general population and among health workers as a global priority (Graham & Brodaty, 1997). Three studies from India (with a mixture of focus group discussion and open-ended interviews) illustrate the pervasive problem in LAMIC (Patel & Prince, 2001; Shaji, Smitha, Praveen Lal, & Prince, 2002b; Cohen, 1995). The typical features of dementia are widely recognized, and indeed named ‘Chinnan’ (literally childishness) in Kerala (Shaji et al., 2002b), ‘nerva frakese’ (tired brain) in Goa (Patel & Prince, 2001) and ‘weak brain’ in Banares (Cohen, 1995). However, in none of these settings was there any awareness of dementia as an organic brain syndrome, or indeed as any kind of medical condition. Rather, it was perceived as a normal, anticipated part of ageing. This general lack of awareness has important consequences:

1. There is little help seeking from formal medical care services (Patel & Prince, 2001).
2. There is no structured training on the recognition and management of dementia at any level of the health service.
3. There is no constituency to place pressure on the government or policy makers to start to provide more responsive dementia care services (Shaji et al. 2002b).
4. While families are the main caregivers, they must do so with little support or understanding from other individuals or agencies.

In the absence of understanding regarding its origins, dementia is stigmatized. In Goa, the likely causes were cited as ‘neglect by family members, abuse, tension and lack of love (Patel & Prince, 2001). In Kerala, it was reported that most caregivers tended to misinterpret symptoms of the disease and to designate these as deliberate misbehaviour by the person with dementia (Shaji et al. 2002b). Sufferers are specifically excluded from residential care, and often denied admission to hospital facilities (Patel & Prince, 2001). Disturbed behaviour, common among people with dementia, is particularly poorly understood leading to stigma, blame, and distress for caregivers (Ferri et al., 2004).

The Madrid Plan (paragraph 44) calls for a strengthening of solidarity through equity and reciprocity between generations. This is unlikely to be achieved without increased awareness and understanding. Intergenerational solidarity is especially important for dementia care. Families are the cornerstone of support for many older people in most LAMIC, particularly those with dementia. 10/66 DRG pilot study data (Choo et al., 2003; Dias et al., 2004; 10/66 Dementia Research Group, 2004) indicated that children or children-in-law were generally the most frequent caregivers for people with dementia. A high proportion of people with dementia in LAMIC live in multigenerational households including young children – many of their carers will have responsibilities for their children as well as their parents. This may be one of the reasons why caregiver strain is as evident as in developed countries, despite extended family networks (Prince, 2004). The 10/66 DRG is planning a series of schools workshops in the communities in which it is working. Children are the future of the community, future carers of older parents and parents-in-law, and future older people. They are receptive, and easily accessed through schools. Social learning theory predicts that insights gained will be passed on to other groups in society – their parents and grandparents, for example (Rahman, Mubbashar, Gater, & Goldberg, 1998). We hope that this model may be generalizable, and of benefit to Alzheimer’s Disease International’s 77 national Alzheimer’s associations.

A critical mass of informed caregivers can assist awareness-raising, provide advice and support to families, and can work with Alzheimer’s associations to lobby for more services that better meet their needs. Community solidarity can effect change through support for policies based on equity and justice – a fairer distribution of healthcare services, and access to effective care regardless of age. Aware communities can provide support, or at least not stigmatize and exclude those with dementia and those who care for them. Policy makers read newspapers and can be held to account by media campaigns backed up by advocacy from committed NGOs. In developed countries dementia awareness is growing rapidly, with the media playing an important part; coverage over18 months in the UK *Daily Telegraph* has increased from 57 articles in 1998/9 (10/66 Dementia Research Group, 2000) to 112 in 2006/7. Recent evidence-based reports from the UK and the Australian Alzheimer’s associations garnered considerable media attention and were instrumental in making dementia a national health priority in both countries. Public awareness in LAMIC is less developed, with few media outlets carrying stories about dementia and ageing - a search in 1999 of the *Times of India* identified no articles (10/66 Dementia Research Group, 2000) 10/66 research teams in Argentina, Venezuela, Peru, Dominican Republic and India have succeeded in getting the message out in newspapers, TV and radio. The *Times of India* published 15 articles in the last 18 months alone. Our experience is that while LAMIC media are receptive to these stories as part of their role in informing the public and stimulating debate, efforts are required to alert them to the importance of ageing and dementia, and to build their capacity to report research and understand its local relevance.

### Social protection in old age

The Madrid Plan (paragraphs 48 and 53) calls for reduction of poverty among older people, and sufficient income for all older people, particularly disadvantaged groups. This call is specifically supported by the UN Charter of Human Rights and the UN Convention on the Rights of Persons with Disabilities, and is also a key Millennium Development Goal. 10/66 DRG pilot studies have shown that people with dementia, and their families are particularly likely to be financially disadvantaged (10/66 Dementia Research Group, 2004). Caregivers often cut back or stop work to care. People with dementia do not receive disability benefits, even in countries where these are theoretically available. There are also no compensatory benefits for caregivers.

Social protection in old age depends upon a complex interaction of health, living arrangements, family support, sources and levels of income. In LAMIC, family support is neither ubiquitous nor comprehensive. In most 10/66 population-based study centres (Table II) we have found that around 5% of older people have no children, lower in China (2.8% to 3.2%) and higher in the Dominican Republic (13%), Peru (10%) and Cuba (15%). A further one fifth of older people have no children living within 50 miles. Those living alone (3%–12% by centre) or only with a spouse (16%–49%) are also potentially vulnerable. Gifts of money from family (family transfers) are an important source of income in 10/66 centres with low pension coverage (Dominican Republic, rural Mexico, rural China and India). While transfers are targeted on those without pensions, not living with children, the physically ill and users of health services, they are also inequitable in that wealthier households are more likely to benefit. In 10/66 centres with high pension coverage (Cuba, Peru, Venezuela and urban China), older people are retired and family transfers are both unnecessary and uncommon. In centres with low pension coverage, a significant minority continue to work and 25% or more are reliant on family transfers; food insecurity (an important index of absolute poverty – going hungry because of lack of money to buy food) was reported by a substantial minority. Data from the 10/66 population-based surveys will be an important resource for improving understanding of the complex links between incomes, pensions, poverty, social protection and health.

**Table II:** The social protection context for older people (aged 65 years and over) in 10/66 catchment area surveys in Latin America, India and China.

The catchment area samples		Sources of income			Vulnerable living arrangements			Vulnerable family support			Poverty	Dependency
					
Population based centre catchment area	*n*	Government or occupational pension %	Family transfers %	Paid work %	Living alone %	Living with spouse only %	Total %	No children %	No children within 50 miles %	Total %	Food insecurity %	Needs for care
Cuba	2944	82.1	8.9	10	8.9	27.3	36.2	14.7	3.6	18.3	4.8	8.7
Dominican Republic	2011	30.4	29	8.2	12.6	21.8	34.4	10.6	9.7	20.3	12.1	11.7
Venezuela	1958	57.5	5.2	5.2	3.1	16.5	19.6	4.5	3.1	7.6	5.9	9.8
Peru (Urban)	1381	65.7	4.9	0.9	3.3	16.8	21.1	10.1	3.2	13.3	4.6	9.8
Peru (Rural)	552	64.7	0.7	1.4	8.0	21.6	29.6	6.9	4.5	11.4	13.5	4.7
Mexico (Urban)	1003	72.7	11.2	7.3	10.6	15.1	25.7	6.3	3.4	9.7	3.9	11.4
Mexico (Rural)	1000	25.4	18.8	12.4	11.2	15.6	26.8	6.3	2.7	9.0	8.6	8.1
China (Urban)	1160	90.5	4.7	0.0	4.7	49.2	53.9	1.6	1.6	3.2	0.0	15.1
China (Rural)	1002	3.8	36.4	0.6	4.9	21.9	26.8	2.5	0.3	2.8	1.2	4.8
India (Urban)	1000	11.6	37.8	2.4	4.4	15.9	20.3	6.2	3.2	9.4	20.8	2.8
India (Rural)	999	34.6	46.8	15.8	12.0	18.7	30.7	5.7	2.6	8.3	14.1	8.5

Some LAMIC governments have sought to encourage or coerce families to shoulder their responsibility for the financial support and care for older parents (Prince et al., 2007a). For example, the Indian parliament passed a law in 2007 requiring children to support their parents, with those who fail to do so facing a three month prison term with no right of appeal. The legislation states ‘old age has become a major social challenge and there is need to give more attention to care and protection of older persons. Many older persons … are now forced to spend their twilight years all alone and are exposed to emotional neglect and lack of physical and financial support.’ The Social Justice Minister, Meira Kumar, said, ‘This bill is in response to the concerns expressed by many members over the fate of the elderly. With the joint family system withering away, the elderly are being abandoned. This has been done deliberately as they (the children) have a lot of resources which the old people do not have.’ The legislation also provides for the state to set up old age homes that the minister said should be the ‘last resort for the poor and the childless.’ While such stop-gap policies are understandable in the context of the very real social problem identified by Indian lawmakers, they seem destined to fail in the longer-term. Inexorable trends towards more internal and international migration, declining fertility, higher levels of education and increased participation of women in the workforce will, inevitably, reduce the availability and willingness of children (principally daughters and daughters-in-law) to care (Prince et al., 2007a).

More sustainable poverty reduction strategies include universal non-contributory social pensions (the focus of a campaign run by HelpAge International – http://www.helpage.org/Researchand policy/Socialprotection), targeted disability pensions and caregiver benefits. For older people in developing countries ‘dependency anxiety’ (Patel & Prince, 2001; Cohen, 1995; Vatuk, 1990) – not wanting to be a burden on relatives, fearing inadequate support, and therefore wishing to maintain independence from the family – is a key motivating principle. Social pensions address these concerns directly, providing insurance against the risks that older people face, including uncertainty over how long they will live, how long they will remain healthy, whether they can count upon the support of others if they need it, and how long they can earn an income. Social pensions play a significant role in alleviating chronic poverty in that that they can support whole families; elderly pensions provide 37% of Brazilian household income, and living with an older person in receipt of a pension is seen to be a key to family financial stability (Garcez-Leme, Leme, & Espino, 2005). Having a pensioner in the family reduces the risk of the household becoming poor by 21% (Institute of Development and Policy Management/HelpAge International, 2003). Older people consistently invest the money they have in income-generating activities and the health and education of dependants; in rural Brazil pensions are strongly associated with increased school enrolment, particularly of teenage girls (Gorman, 2004). Most importantly they serve to reinforce reciprocal family ties, changing the perspective from one in which older people are seen as a dependent drain upon household resources to one in which they can be properly valued for their non-economic as well as their economic contributions. Dependent older people would be particularly likely to benefit - informal care would be bolstered and formal/paid care would be more affordable.

### Access to age-appropriate healthcare

The Madrid Plan (paragraph 74) calls for the elimination of social and economic inequalities in access to healthcare and the development of primary, secondary healthcare and long-term care to meet the needs of older persons (paragraphs 75–77). Primary health care services in LAMIC fail older people with dementia (Patel & Prince, 2001; Shaji et al., 2002b; Dias et al., 2004; Prince, Livingston & Katona, 2007b) as they focus on acute ‘treatable’ conditions and are entirely clinic based. There is a need for training in the basic curriculum regarding diagnostic and needs based assessments, and a paradigm shift beyond the current preoccupation with simple curative interventions to encompass long-term support and chronic disease management. Given the frailty of many older people with chronic health conditions, there is also a need for outreach care, assessing and managing patients in their own homes. The WHO has proposed an alternative, feasible, generalizable care framework (Innovative Care for Chronic Conditions – ICCC) focusing upon the need for:

1. a dialogue to build consensus and political commitment for change,
2. a paradigm shift towards extended, regular healthcare contact,
3. an integrated multi-sectoral approach,
4. centring care on patients and families,
5. supporting patients in the community,
6. an emphasis on prevention.

While this model notably makes no mention of dementia, it is likely to be as applicable for this condition as for those other chronic non-communicable diseases – mental disorders, stroke, heart disease and diabetes – for which it was initially proposed. While training of community health staff can be promoted through changes to the medical and nursing school curricula, sustainable reorientation/ reorganization of basic services for older people will need new government policy.

10/66 population-based study data suggest that barriers to accessing healthcare among older people in LAMIC include poverty, frailty, low levels of education and older age. People with dementia and their families are particularly unlikely to access healthcare, despite the high levels of associated disability and caregiver strain. Lack of demand for services arises in part from a tendency to view dementia as a normal part of ageing. Encouraging help-seeking requires community dissemination to increase awareness with information from government, healthcare providers and media. However, efforts to increase demand must be accompanied by health system and service reform, so that help-seeking is met with a supply of better-prepared, more responsive services, such as those proposed in WHO ICCC. 10/66 is testing the effectiveness of training community healthcare workers to identify people with dementia (Shaji, Arun Kishore, Lal, & Prince, 2002a; Ramos-Cerqueira et al., 2005; Jacob, Senthil, Gayathri, Abraham, & Prince, 2007), and to deliver a brief intervention to educate and train caregivers (Prince et al., 2007a). In practice, such interventions will need to be incorporated into horizontally constructed programmes addressing the generic needs of frail, dependent older people and their caregivers, whether arising from cognitive, mental or physical disorders.

The role of specialists within the wider health system requires careful consideration and planning. Most LAMIC have insufficient specialists (psychiatrists, neurologists, elderly care physicians) dedicated to dementia care to provide frontline dementia services nationwide. Among the countries involved in the 10/66 research programme, only Venezuela (24 psychiatrists/100,000 population), Argentina (13/100,000) and Cuba (11/100,000) have resources similar to those in developed countries. Other Latin American countries, China, India and Nigeria (2, 1, 0.2 and 0.1/100,000) are much less well resourced. The Madrid Plan suggests a broader role for specialists in:

- training healthcare professionals, ensuring early assessment and diagnosis
- providing community programmes to support people at home
- providing respite care for patients and carers
- promoting public information about the symptoms, treatment, consequences and prognosis

### Disability, dependency and long-term care

Disability impacts mainly upon older people, who are particularly likely to have multiple physical, mental and cognitive disorders. Demographic ageing and the health transition (increasing morbidity from chronic non-communicable diseases) will inevitably mean large increases in their numbers in coming years. An adequate response to these challenges will require (a) policies to prevent disability through the control of NCDs, (b) policies to limit disability through more active community-based rehabilitation, (c) policies to mitigate the effects of disability upon participation, and (d) policies to manage disability through universal access to long-term care. Such measures are already strongly advocated through international agreements. The Madrid Plan (paragraph 90) calls for the maintenance of maximum function, and the fullest possible societal participation of older persons with disabilities. The UN Convention on the Rights of People with Disabilities (2006) enshrines participation, income and access to healthcare as basic rights for all disabled people.

In the 10/66 population based studies, we have found that 5–10% of older people need care, the responsibility falling mainly on female family members. The WHO report on long-term care policy (WHO, 2000) notes wide variation in the responsibilities of individuals, families and the state, but considers that each community could and should determine transparently the types and levels of assistance needed by older people and their carers, and the eligibility for and financing of long-term care support. In practice, LAMIC governments typically eschew involvement in providing or financing long-term care (Prince et al., 2007a), and few if any have comprehensive policies and plans.

Using epidemiological data to promote understanding of the societal costs of disability, and the relative contributions of different NCDs should inform prioritization – and a shift towards primary prevention and better long-term care provision for those with disability.

## Conclusion

There is an urgent need for much more epidemio-logical research to assist in raising awareness of the unmet needs of the rapidly growing number of older people with dementia and other chronic non-communicable diseases, living in low and middle income countries. Many have disabilities and need long-term care, currently provided by family carers. However, family support is not always available. Primary care services do not meet their needs. Governments neither provide long-term care nor support carers. Many older people are financially vulnerable, particularly in countries with low pension coverage. Disability is linked to poverty, and imposes economic strain on families.

For our part, the 10/66 Dementia Research Group, partnered by Alzheimer’s Disease International and other NGOs has developed a detailed dissemination strategy using findings from population-based studies involving 21,000 older people in nine LAMIC to engage with public and policy makers in Latin America, India, China and Africa. We aim to use community workshops, schools engagement, oral testimonies and films to raise awareness and stimulate debate about older people’s needs and how these should be addressed. We will distribute newsletters, policy analyses, briefings and reports, and redesign our website (http://www.alz.co.uk/1066) with interactive content for participants, public, policy makers and researchers. Our interactive engagement programme – bringing together older people, their carers, local communities, researchers, stakeholders, opinion formers, advocates, media and policy makers – will advance the vision of the Madrid International Plan for Action on Ageing for ‘a society for all ages’.
